# Reducing Inappropriate Proton Pump Inhibitors Use for Stress Ulcer Prophylaxis in Hospitalized Patients: Systematic Review of De-Implementation Studies

**DOI:** 10.1007/s11606-020-06425-6

**Published:** 2021-02-02

**Authors:** Claudia C. Orelio, Pauline Heus, Judith J Kroese-van Dieren, René Spijker, Barbara C. van Munster, Lotty Hooft

**Affiliations:** 1 Cochrane Netherlands, Julius Center for Health Sciences and Primary Care, University Medical Center Utrecht, Utrecht University, Utrecht, The Netherlands; 2 Research Support, Diakonessenhuis Utrecht, Utrecht, The Netherlands; 3 Julius Center for Health Sciences and Primary Care, University Medical Center Utrecht, Utrecht University, Utrecht, The Netherlands; 4 University Medical Center Groningen, University Center for Geriatric Medicine, University of Groningen, Groningen, The Netherlands

**Keywords:** proton pump inhibitor (PPI), de-implementation, systematic review, stress ulcer prophylaxis (SUP), hospital

## Abstract

**Background:**

A large proportion of proton pump inhibitor (PPI) prescriptions, including those for stress ulcer prophylaxis (SUP), are inappropriate. Our study purpose was to systematically review the effectiveness of de-implementation strategies aimed at reducing inappropriate PPI use for SUP in hospitalized, non-intensive care unit (non-ICU) patients.

**Methods:**

We searched MEDLINE and Embase databases (from inception to January 2020). Two authors independently screened references, performed data extraction, and critical appraisal. Randomized trials and comparative observational studies were eligible for inclusion. Criteria developed by the Cochrane Effective Practice and Organisation of Care (EPOC) group were used for critical appraisal. Besides the primary outcome (inappropriate PPI prescription or use), secondary outcomes included (adverse) pharmaceutical effects and healthcare use.

**Results:**

We included ten studies in this review. Most de-implementation strategies contained an educational component (meetings and/or materials), combined with either clinical guideline implementation (*n* = 5), audit feedback (*n* = 3), organizational culture (*n* = 4), or reminders (*n* = 1). One study evaluating the de-implementation strategy effectiveness showed a significant reduction (RR 0.14; 95% CI 0.03–0.55) of new inappropriate PPI prescriptions. Out of five studies evaluating the effectiveness of de-implementing inappropriate PPI use, four found a significant reduction (RR 0.21; 95% CI 0.18–0.26 to RR 0.76; 95% CI 0.68–0.86). No significant differences in the occurrence of pharmaceutical effects (*n* = 1) and in length of stay (*n* = 3) were observed. Adverse pharmaceutical effects were reported in two studies and five studies reported on PPI or total drug costs. No pooled effect estimates were calculated because of large statistical heterogeneity between studies.

**Discussion:**

All identified studies reported mainly educational interventions in combination with one or multiple other intervention strategies and all interventions were targeted at providers. Most studies found a small to moderate reduction of (inappropriate) PPI prescriptions or use.

**Supplementary Information:**

The online version contains supplementary material available at 10.1007/s11606-020-06425-6.

## INTRODUCTION

Proton pump inhibitors (PPIs) reduce the production of gastric acid and are used for the treatment of a variety of gastrointestinal (GI) disorders, as well as for stress ulcer prophylaxis (SUP)^1–3^. Stress ulcers can develop in hospitalized patients who are exposed to physiological stress conditions or due to polypharmacy^2,4^. In a small percentage of patients, stress ulcers result in clinically important GI tract bleeding (CIB)^2,4^. SUP prevents ulcer development, and can decrease bleeding incidence^5^.

Patient risk factors for CIB include coagulopathy, chronic liver disease/hepatic failure, male gender, sepsis, shock, previous GI bleeding, or kidney failure. Drug-related risk factors include high-dose corticosteroids, non-steroidal anti-inflammatory drugs (NSAID), or anticoagulant use^4–8^. CIB in hospitalized, non-ICU patients is not well investigated, but reported incidence is found to be low (0.2–0.4%)^4,9^. In line with this, several guidelines do not recommended SUP for non-ICU patients without additional risk factors^2,10^.

Despite this advice against SUP prescription in non-ICU patients, the use of SUP, especially PPIs, in hospitalized patients has steadily increased worldwide^11–14^. With a limited group of patients at risk of developing CIB due to stress ulcers, the benefit of SUP^4,6,9^ is over-estimated and prescribed too often^1,2,4,14–16^. Additionally, many patients upon hospital admission are already inappropriately using PPIs, and SUP prescriptions are inappropriately continued upon discharge^1,14,15^. As PPIs may interact with other drugs and have potential adverse side effects, these patients are exposed to unnecessary health risks, (e.g., *Clostridium difficile* infections and pneumonia, increased risk of osteoporotic fractures, increased mortality)^3,9,17–19^ (Suppl. Table 1). In addition, the healthcare system is confronted with unnecessary costs^14,15^ and the pharmaceutical residues in waste and surface water contribute to environmental pollution and are associated with health risks^20,21^. Medical drug usage review during a hospitalization period provides an opportunity for de-prescription.

Over the last years, several countries (e.g., USA, China, The Netherlands) have initiated campaigns to decrease inappropriate medical treatments, referred to as low-value care^14,22,23^. The goals of these campaigns were to (1) improve healthcare quality by prevention of unnecessary health risks, and (2) restrain healthcare costs^22,24^. In the ageing population with increasing patient numbers with multimorbidity, and related polypharmacy, this is even more important. Inappropriate prescriptions of SUP are recognized as low-value care by the Society of Hospitals and included in their Choosing Wisely campaign^23^. Their call for action has urged healthcare providers to construct and implement interventions to reduce the inappropriate use of SUP.

In order to change clinical care and drug prescriptions, many possible strategies have been described (educational, feedback and communication interventions, financial incentives to change prescription behavior of healthcare providers, patient attitude changes)^22,24–27^.

While studies that investigated the incidence of inappropriate SUP in individual institutions are abundant, reporting of interventional strategies to reduce inappropriate PPI use in hospitalized, non-ICU patients is limited. The purpose of this systematic review was to identify and compare strategies that have been used to reduce inappropriate PPI use for SUP in adult, hospitalized, non-ICU patients.

## METHODS

We followed Cochrane guidelines in conducting this review and report it following the Preferred Reporting Items for Systematic Reviews and Meta-analyses (PRISMA) statement^28,29^. This protocol was registered in PROSPERO (CRD42020165508).

### Study Identification

EMBASE (Ovid) and Medline (Ovid) electronic databases were searched by an information specialist (RS) on the 8th of January 2020 from inception, without restrictions on publication date or language. Searching included indexing terms, free text terms, and synonyms for proton pump inhibitor combined with terms for low-value care and hospitalized patients (detailed information in Supplementary Table 2a/b). Expert in the field (BvM) retrieved one study reference independently from the systematic literature search.

### Selection of Studies

We included studies of adult, hospitalized patients in non-ICU settings, in which an intervention to reduce the use of inappropriate PPI was evaluated. (Quasi-) randomized controlled trials and comparative observational studies reported in English, Dutch, or German were eligible for inclusion. Studies that addressed both PPI and H2RA medication as SUP were included if data on PPI use could be extracted separately. Studies combining inpatient and outpatient data were included when inpatient data could be extracted separately.

Pairs of authors (CO, PH, JJKvD) independently screened all titles and abstracts that were retrieved from the literature search, using Rayyan Software^30^. Subsequently, they assessed final eligibility based on full-text assessment. Disagreements between the authors were resolved by discussion.

### Data Extraction and Critical Appraisal

Data were extracted by one author (CO/JJKvD) and checked by another author (PH). Disagreements were resolved by discussion or involvement of a third author (LH). A predefined, piloted digital form was used for data extraction (including details of study design, participants, setting, de-implementation strategies (components and targets), outcomes). The interventions used for de-implementation were classified based on the taxonomy provided by the Cochrane Effective Practice and Organisation of Care (EPOC) Group (Suppl. Table 3)^31^. Four categories of target audiences were distinguished: healthcare providers, patients, organization, and system. Besides our primary outcome (inappropriate PPI prescription or use), secondary outcomes of interest included pharmaceutical effects (symptoms of acid reflux; ulcer and upper gastrointestinal bleeding), adverse pharmaceutical effects (diarrhea or obstipation, abdominal pain, *Clostridium difficile* infections, hospital-acquired pneumonia, electrolyte disturbances), and healthcare use (e.g., length of stay (LOS), ICU or hospital admission, emergency department visit, alternative medication use). Two authors (PH, CO or JJKvD) independently assessed the risk of bias (RoB) using suggested criteria for EPOC reviews^31^.

### Analysis

Descriptive characteristics of studies were summarized narratively. To quantify the effectiveness of de-implementation strategies, we calculated the proportion of inappropriate PPI prescriptions or inappropriate PPI use for each study arm. PPI inappropriateness was defined as no indication for SUP in patients without risk factors, but definitions of indication for SUP differed between included studies.

We distinguished two groups of patients: one consisted of patients who were using PPI medication prior to hospitalization that was continued during hospitalization and the second group were patients who started PPI medication during hospitalization. To translate this distinction in patient groups to different categories of PPI medication application, we defined PPI use as all PPI prescriptions during hospitalization (i.e., continued or new PPI prescriptions). We defined PPI prescriptions as PPI prescriptions that started during hospitalization.

Results for the primary outcome are presented in forest plots that were generated with Review Manager (RevMan5.3) software. Meta-analysis revealed considerable heterogeneity between studies (based on visual inspection of forest plot and *I*^2^ > 50%). Therefore, a pooled effect estimate was not presented. The results for the secondary outcomes of interest were described narratively. Funnel plot analysis, to address the issue of publication bias, was not performed, because too few studies for a similar outcome were retrieved.

## RESULTS

### Search and Screening Results

**Figure 1:**
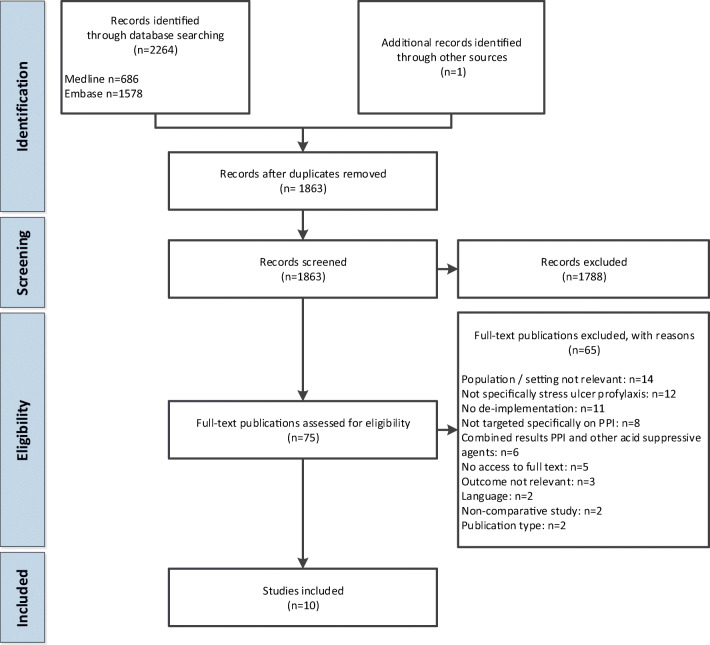
**Flowchart of study selection**.

We retrieved 2264 studies from database searches and one study through experts in the field. After duplicate removal, 1863 studies remained, of which 75 were selected for full-text review (Fig. 1). Main reasons for excluding studies were non-relevant population or setting and when authors indicated that de-implementation was not directed at reduction of PPI for SUP. Finally, ten studies were included in the analyses.

### Characteristics of Included Studies

The studies were published from 1998 to 2018 and all, but one, had a before-after study design without a parallel control group. They were mainly conducted in high- and middle-income countries and as single-center studies (Table 1).

**Table 1:** Characteristics of the Included Studies

Source	Country	Single/multicenter	Hospital	Study design
del Giorno 2018	Switzerland	Multi	General; academic	Non-randomized trial
Jain 2013	USA	Single	General	Before-after
Kehr 2011	USA	Single	General	Before-after
Khalili 2010	Iran	Single	General	Before-after
Khudair 2011	Qatar	Single	Academic	Before-after
Kumana 1998	Hong Kong	Single	General	Before-after
Luo 2018	China	Single	General	Before-after
van Vliet 2009	Netherlands	Multi	General; academic	Before-after
Xin 2018	China	Single	General	Before-after
Yachimski 2010	USA	Single	Academic	Before-after

#### Population and Setting

A number of studies were performed at specific non-ICU hospital departments, such as the internal medicine department^32^, general ward^33^, infectious disease ward^34^, or a pulmonary medicine ward^35^ (Table 2). In other studies, various departments were included in the study and either ICU participants were excluded^36,37^, or data for non-ICU participants were separately reported^38,39^. Two studies included multiple departments and did not report exclusion of ICU participants^13,40^. However, based on their indications for PPI prescription, we assumed no ICU participants were included.

**Table 2:** Patient Characteristics of the Included Studies

Source	Departments at which study was conducted	Number	Participants	Gender (% female)
			Age (mean age years ± SD)	
del Giorno 2018	Internal medicine, surgery	i: 26,312 c: 18,661	i: 75 (63–83) c: 67 (50–78)^§^	i: 50 c: 52
Jain 2013	Internal medicine	b: 54 a: 49	Not specified	Not specified
Kehr 2011	Family medicine inpatient service*	b: 59 a (1 m): 51 a (4 m): 46	b: 58 a (1 m): 64 a (4 m): 59	b: 38 a (1 m): 23 a (4 m): 28
Khalili 2010	Infectious disease	b: 265 a: 241	> 10 years	b: 46 a: 49
Khudair 2011	General medical	b: 206 a: 208	b: 51 ± 17 a: 53 ± 19	b: 19 a: 34
Kumana 1998	All departments**	b: 173 a: 546	Not specified	Not specified
Luo 2018	> 10 departments**	b: 300 a: 300	b: 51 ± 14 a: 49 ± 15	b: 39 a: 45
van Vliet 2009	Pulmonary medicine	b: 300 a: 300	b: 58 ± 17 a: 56 ± 16	b: 45 a: 48
Xin 2018	7 departments	b: 142 a: 143	b: 58 ± 14 a: 59 ± 14	b: 58 a: 59
Yachimski 2010	Not specified, ICU excluded	b: 458 a: 484	b: 63 ± 19 a: 63 ± 18	b: 41 a: 43

*i*, intervention group; *c*, control group; *b*, before de-implementation; *a*, after de-implementation; *m*, months

*Including ICU

**No details provided whether ICU was included

^§^Median age (IQR)

#### Appropriateness of PPI for SUP

Authors of the included studies based their definition of appropriateness of PPI use for SUP (Suppl. Table 4) on a variety of (inter)national guidelines and available literature (Suppl. Table 5). Four studies referred to the 1999 ASHP guideline^10^, in which acid-suppressive therapy for low-risk non-ICU hospitalized patients is advised against. In one study, a multidisciplinary team reviewed literature to establish an institutional guideline^36^, and in another study, relevant specialists established criteria for appropriate PPI use^40^.

#### De-Implementation Strategies

One study identified barriers and facilitators to reducing the use of PPI prior to inform the design of the de-implementation strategy^32^. Three other studies referred to literature for effective de-implementation or teaching strategies^35,37,40^ (Table 3; Suppl. Table 5).

**Table 3:** Characteristics of the De-Implementation Strategies

Source	Intervention(s)^§^	Target(s)	Intervention(s) provided by	Barriers and facilitators identified prior to intervention
del Giorno 2018	Educational meetings, educational materials, clinical practice guideline, audit and feedback, local opinion leaders	Provider: medical staff	Not specified	No*)
Jain 2013	Educational meetings, organization culture (discussion during morning rounds)	Provider: medical staff	Not specified	Yes
Kehr 2011	Educational meetings, educational materials	Provider: medical staff	Medical and pharmacy staff	No
Khalili 2010	Educational meetings, clinical practice guideline	Provider: medical staff	Pharmacy staff	No
Khudair 2011	Educational materials, reminders, audit and feedback, clinical practice guideline, organizational culture (multidisciplinary rounds)	Provider: medical and pharmacy staff	Medical and pharmacy staff	No
Kumana 1998	Educational meetings, educational materials, audit and feedback	Provider: medical staff	Not specified	No*)
Luo 2018	Clinical practice guideline, organization culture (pharmacist-led reward and punishment mechanism)	Provider: medical staff	Pharmacy staff, management	No
van Vliet 2009	Educational meetings, education materials, clinical practice guideline, organization culture (discussion during grand rounds)	Provider: medical staff	Not specified	No*)
Xin 2018	Educational meetings, educational materials, inter-professional education	Provider: medical staff	Pharmacy staff	No
Yachimski 2010	Educational meetings, educational materials, clinical practice guideline	Provider: medical staff	Pharmacy staff	No

^§^Classification based on EPOC (1)

*) Authors refer to literature for most effective implementation strategies

In all but one, de-implementation strategies contained an educational component (meetings and/or materials), which was combined with implementation of a clinical practice guideline in five studies^33–37^, reminders in one study^33^, audit feedback in three studies^33,37,40^, an organizational culture intervention in three studies^32,33,35^, and an inter-professional education in one study^39^. The one study without an educational intervention combined implementation of a clinical practice guideline with an organizational intervention^13^.

In all studies, the de-implementation strategy targeted the medical staff, and one study targeted both pharmacy and medical staff^33^ (Table 3). The educational components of de-implementation strategies were provided by pharmacy staff^34,36,39^, pharmacy staff in combination with medical staff^33,38^, or management^13^. In four cases, it was not specified who provided the de-implementation strategy.

### Critical Appraisal

**Figure 2:**
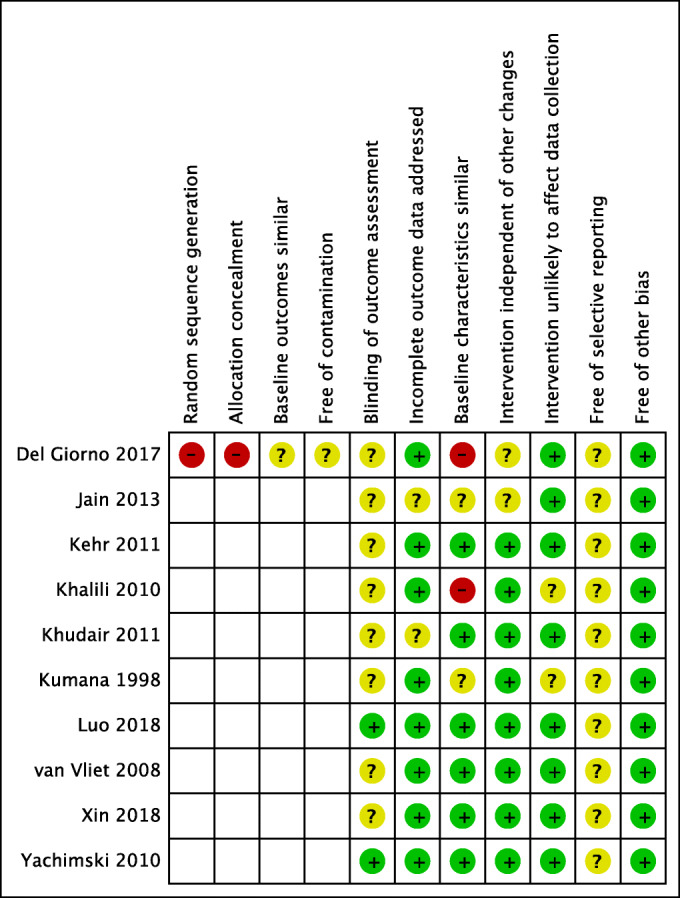
**Summary of the risk of bias assessment**.

The summary of the risk of bias (RoB) assessment is presented in Fig. 2, and substantiation for RoB judgement is presented in Supplementary Table 6.

The study of Del Giorno was the only trial which included a control group and therefore four additional RoB items were assessed^37^. As this study was a non-randomized trial, we scored RoB for random sequence generation and allocation concealment at “high risk.” The interventions were conducted at the internal medicine departments of five hospitals, with the surgery departments in the same hospitals serving as control. As a consequence, the participant population at baseline were dissimilar, but this was not further addressed by the authors. Also, the interventions conducted at one department could have potentially contaminated the results at the control department within the same hospital. Therefore, both these items were scored “unclear risk” of bias.

Most studies scored “unclear risk” of bias for blinding of outcome assessment as they did not report how outcome assessment was performed^32–35,37–40^. Only two studies scored “low risk” of bias as outcome assessment was automated or standardized^13,36^. Other studies were scored “unclear” risk of bias for blinding of outcome assessment. Six studies scored “low risk” for similar baseline characteristics as participant population baseline characteristics were comparable^13,33,35,36,38,39^. Two studies scored “unclear risk” as no information about the baseline characteristics of participants was reported^32,40^. Two studies score “high risk” as large differences between the two participant groups were observed^34,37^.

### Effectiveness of Interventions

#### Primary Outcome: PPI Use or Prescriptions

**Figure 3:**
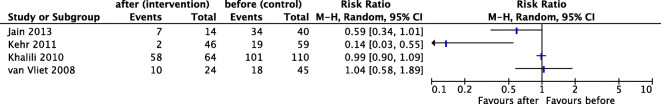
**Forest plot of meta-analysis outcome inappropriate PPI prescriptions. Forest plot of comparison: De-implementation strategy (intervention) versus usual care/no de-implementation (control), outcome: Inappropriate prescriptions during hospitalization. We refrained from calculating a pooled effect estimate, as heterogeneity between studies was large (*I***^**2**^ **= 87%)**.

Four studies addressed inappropriate initiation of PPIs (PPI prescriptions) during hospitalization^32,34,35,38^; data and calculated RRs are presented in a forest plot (Fig. 3). We refrained from calculating a pooled effect estimate, as heterogeneity between studies was large (*I*^2^ = 87%). One study reported a decrease of inappropriate acid-suppressive therapy (RR 0.14; 95% CI 0.03–0.55)^38^. The other three studies showed a non-significant small^32^ or no reduction of inappropriate PPI prescriptions after interventions^34,35^.

**Figure 4:**
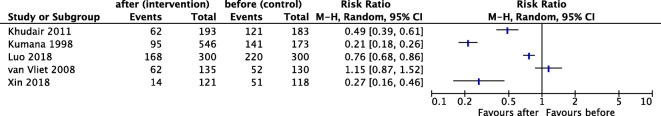
**Forest plot of meta-analysis outcome inappropriate PPI use. Forest plot of comparison: De-implementation strategy (intervention) versus usual care/no de-implementation (control), outcome: Inappropriate use during hospitalization. We refrained from calculating a pooled effect estimate, as heterogeneity between studies was large (*I***^**2**^ **= 97%). (1) EffectivePracticeandOrganisationofCare(EPOC). EPOC Taxonomy.** epoc.cochrane.org/epoc-taxonomy**. 2015. 2020**.

Five studies addressed the outcome inappropriate continuation of outpatient PPI use^13,33,35,39,40^. Also here, data are presented in a forest plot (Fig. 4). One study reported a decrease of acid-suppressive therapy, including a specified proportion of PPI^33^). For this study, numbers of inappropriate PPI were adjusted accordingly. Four studies revealed a significant reduction of inappropriate PPI use after de-implementation, with two studies revealing a moderate to large reduction (RR 0.21; 95% CI 0.18–0.26 and RR 0.27; 95% CI 0.16–0.46)^33,40^ and two studies showing a small reduction (RR 0.49; 95% CI 0.39–0.61 and RR 0.76; 95% CI 0.68–0.86)^13,39^. One study did not reveal a reduction of inappropriate PPI use^35^. We refrained from calculating a pooled effect estimate, as heterogeneity between studies was large (*I*^2^ = 97%).

Three studies (also) reported the outcome PPI prescriptions and/or use without making the distinction between inappropriate versus appropriate PPI for SUP^35–37^. One of these specifically addressed new PPI prescriptions during hospitalization which were continued at discharge^37^. This study reported a slight reduction of new PPI prescriptions in their intervention group (18% at baseline versus 16% post-intervention), while an increase was observed in the control group (30% at baseline versus 36% post-intervention). The other studies compared the PPI prescriptions and use during hospitalization and at discharge^35,36^. A significant difference was observed in PPI prescription (i.e., new prescriptions) during the hospitalization period and at discharge. In contrast, no significant difference was noted in the PPI use (i.e., all PPI prescriptions) during the hospitalization period and at discharge.

Studies differed in many (clinical) aspects, which has contributed to the observed heterogeneity. As there was no clear pattern detected to explain heterogeneity, we refrained from further sensitivity analyses. Also, we did not observe a correlation between the effect size of inappropriate PPI use or prescription and the amount or types of de-implementation interventions in the studies.

#### Secondary Outcomes: (Adverse) Pharmaceutical Effects, Healthcare Use, and Costs

Overall, secondary outcomes were scarcely and inconsistently reported. One study reported that no difference between the pre-intervention and post-intervention period was observed in new or relapses of GI symptoms 3 months after hospitalization (3% versus 2% of the patients)^35^. Two studies reported adverse pharmaceutical effects. Del Giorno et al. reported that admission for and diagnosis of GI bleeding during hospital stay did not increase significantly during the study^37^. Xin et al. reported that several adverse effects (specified in *C. difficile* infections, respiratory infections, hypomagnesemia, adverse skeletal muscle effects, psychiatric symptoms) decreased significantly (35% control group versus 8% intervention group)^39^.

Three studies reported on healthcare use, specifically on length of stay (LOS). No significant differences were reported between the pre-intervention and post-intervention period. All five studies that reported on PPI or total drug costs (expenditure or cost-saving) reported a cost reduction.

## DISCUSSION

In 2013, the Choosing Wisely campaign has identified PPI and H2RA acid-suppressive therapy for SUP as low-value care that should be avoided^23^. We identified ten studies evaluating the effectiveness of strategies to reduce inappropriate PPI use for SUP in adult hospitalized, non-ICU patients. Altogether, we can conclude that small to moderate reductions in inappropriate PPI prescriptions or use can be accomplished in a wide range of hospital settings upon implementation of PPI-reducing strategies. Nevertheless, these results should be interpreted cautiously as the type of study design of most studies (before-after design) has intrinsic limitations (no control group, no randomization, contamination issues). Taking these shortcomings in study design into account, critical appraisal of the quality of included studies revealed moderate quality for most studies (Fig. 2).

Inter-study heterogeneity hampered meta-analysis of the data. The included studies were different regarding several aspects, namely combinations of interventions, type of hospital departments, and hence patient populations, setting (academic or general hospitals), and country. Adding to clinical heterogeneity, the included studies applied different PPI prescription/use appropriateness criteria (Suppl. Table 4), and based these criteria on a variety of information (Suppl. Table 5). Consequently, studies differed in the indications and specifications of symptoms in which PPI prescription was considered appropriate, including several gastrointestinal tract indications (e.g., reflux disease, peptic ulcer disease) and SUP for high-risk patients^13,33,35,37,39,40^ (Suppl. Table 4). Finally, lack of reporting standardization impaired data extraction and analysis.

Evidence on prevention of GI bleeding by PPI in low-risk patients has been lacking for a long time, and recently, it was shown that even in ICU patients at risk of GI bleeding, PPI prescription did not prevent CIB occurrence^41^. Despite clear advice against SUP prescription for non-ICU-hospitalized patients without additional risk factors in guidelines, this recommendation is apparently not followed by healthcare professionals. It is questionable whether an update of the guideline with evidence is needed or that stricter adherence to guidelines is required. Nevertheless, the ASHP guideline is based on outdated information, with evidence for CIB prevention coming from studies when H2RAs were more commonly used. Also, most studies examined short-term SUP use, while nowadays patients are on continued PPI prescription of which long-term adverse effects are not thoroughly examined.

### Clinical Implications

In agreement with other systematic reviews addressing effectiveness of interventions to change healthcare, we observed large heterogeneity between studies and outcomes, in combination with low study design quality^42–44^. Also, the number of interventions in the included studies was limited mostly to educational interventions directed at providers. None of the studies targeted interventions at patients, even though patient participation in the reduction of inappropriate PPI prescriptions has been shown effective^25,45–47^. Combinations of educational interventions, a reflective practice and supportive environment, are required for high-value healthcare^27,48^. None of the studies included e-health solutions, while computer-assisted decision aids and web-based information for both patients and medical professionals could be instrumental in de-implementing PPI^49^.

Additionally, none of the studies provided sufficient intervention details to allow knowledge transfer of effective intervention strategies. De-implementation, giving up a clinical behavior, has repeatedly been shown to be psychologically more challenging than adopting a new behavior^22,50^. Therefore, it should be acknowledged that de-implementation strategies to change the prescription behavior of healthcare providers require thorough analysis of the local clinical setting and identification of barriers and facilitators^22,51^. This prior analysis was minimally addressed and reported in the included studies.

Future de-implementation studies should take all the contextual factors into account when designing a strategy, and standardization in data collection and reported outcomes would further improve knowledge transferability. Subsequently, for impact evaluation of the de-implementation strategies, authors need to collect and report all essential information needed to interpret and apply their results into practice. This includes knowledge on barriers and facilitators, de-implementation strategy details, sustainability of observed effects, and insight into unintended consequences of the de-implementation strategy. There are several relevant reporting guidelines that can assist authors^52–54^. Finally, the field of de-implementation science in healthcare would benefit from high-quality studies with more rigorous study designs (i.e., cluster RCTs, interrupted time series studies, etc.) that adhere to international reporting guidelines and recommendations on implementation strategy classification. Altogether, we recommend that future de-implementation studies of clinical pathways involve a multidisciplinary approach in which clinicians collaborate with patient representatives and facilitatory services in their organization (i.e., communication, finance, education departments) to ensure that all aspects of an effective implementation are addressed and are supported throughout the organization.

### Strengths and Limitations of This Review

The strength of this systematic review is the focus on the reduction of inappropriate PPI prescriptions/use in hospitalized patients in non-ICU settings. This is not often specifically addressed, and is justified by the increased popularity of PPI use in non-ICU-hospitalized patients^11–14^ and several reports on adverse effects attributed to long-term PPI use^3,9,17–19^.

A limitation of this review is that terminology to describe de-implementation strategies varies widely. In an effort to retrieving all relevant studies, we applied an extensive search strategy. Nevertheless, we cannot exclude the possibility that we missed relevant studies.

## CONCLUSIONS

Among ten studies aimed at reducing inappropriate PPI use for SUP in adult, hospitalized, non-ICU patients, all used mainly educational intervention strategies targeted at providers. Some studies had a small to moderate reduction of inappropriate PPI prescriptions or use. No specific de-implementation intervention was identified as being superior. The studies were heterogenous, due to differences in study populations, settings, reported outcome measures, and combinations of interventions. In general, there was poor reporting and implementation design.

## Supplementary Information

ESM 1 (DOCX 196 kb)
