# Supplementary material for: Reducing Inappropriate Proton Pump Inhibitors Use for Stress Ulcer Prophylaxis in Hospitalized Patients: Systematic Review of De-Implementation Studies
Source: J Gen Intern Med. 2021 Feb 2;36(7):2065–73. doi: 10.1007/s11606-020-06425-6 (PMC8298652; doi:10.1007/s11606-020-06425-6)
Supplement: Supplementary file 1 — (DOCX 196 kb) [file 11606_2020_6425_MOESM1_ESM.docx]

**Orelio et al. Supplementary Tables**

**Supplementary Table 1. PPI adverse patient health effects according to included studies**

| **Adverse effects main categories** | **Adverse effects** | **Reference** |
| --- | --- | --- |
| Gastrointestinal effects | - Enteric infections (*C. difficile*, *Campylobacter*)  - Atrophic gastritis | ([1-7](#_ENREF_1)) |
| Malabsorption of minerals and vitamins | - Hypomagnesaemia  - vitamin B12 reduced absorption/ insufficiency  - Osteoporosis/ bone fractures | ([1-3](#_ENREF_1), [5-7](#_ENREF_5)) |
| Kidney disease | - Interstitial nephritis/ deteriorating kidney function | ([1](#_ENREF_1)) |
| Other associations of unclear significance | - Community-acquired pneumonia (CAP)  - Increased risk for re-hospitalization (and additional avoidable health care costs)  - Increased risk of dementia | ([1-7](#_ENREF_1)) |
| Adverse drug-drug interactions (i.e. clopidogrel) |  | ([2](#_ENREF_2), [5](#_ENREF_5), [7](#_ENREF_7)) |

**Supplementary Table 2a. MEDLINE search strategy sorted by search number ascending**

| **Ovid MEDLINE(R) ALL <1946 to January 07, 2020>A:D** | | |
| --- | --- | --- |
| **#** | **Searches** | **Results** |
| 1 | proton pumps/ai or exp Proton Pump Inhibitors/ or rabeprazole/ or omeprazole/ or ((proton-pump adj inhibitor?) or omeprazol?).ti,ab. or (rabeprazol? or pantoprazol? or lansoprazol? or esomeprazol? or antgra or audazol or aulcer or belmazol or ceprandal or danlox or demepraxol or desec or dizprazol or dudencer or elgam or emeproton or epirazole or erbolin or exter or gasec or gastrimut or gastroloc or gibancer or indurgan or inhibitron or inhipump or lensor or logastric or lomac or losec or mepral or miol or miracid or mopral or morecon or nilsec or nopramin or amep or omp or omz or ocid or olexin or omapren or omed or omegast or omepral or omeprazol or omeprazole or omeprazolum or omeprazon or omeprol or omesek or omezol or omezolan or omid or omisec or dakar or omizac or ompanyt or ortanol or osiren or ozoken or paprazol or parizac or pepticum or pepticus or peptilcer or prazentol or prazidec or prazolit or prilosec or procelac or proclor or prysma or ramezol or regulacid or sanamidol or secrepina or tedec ulceral or ulceral or ulcesep or ulcometion or ulcozol or ulcsep or ulsen or ultop or ulzol or victrix or zefxon or zegerid or zepral or zimor or zoltum or zanprol or ufiprazole or ufiprazol or ufiprazolum or nexium or perprazole or nexiam or inexium or sompraz or axagon or esopral or lucen or axiago or agopton or alexin or amarin or aprazol or bamalite or blason or compraz or estomil or fudermex or gastrex or gastride or gastroliber or ilsatec or ketian or keval or lancid or lanfast or lanproton or lansopep or lansoprazolum or lansox or lanston or lanz or lanzo or lanzogastro or lanzol or lanzopral or lanzor or lasoprol or limpidex or lizul or mesactol or monolitum or ogast or ogasto or ogastro or opiren or pampe or peptomil or prevacid or prezal or promp or prosogan or suprecid or takepron or ulcertec or uldapril or ulpax or unival or zoprol or zoton or pantoprazole or pantoprazol or pantoprazole or pantoprazolum or controloc or pantoloc or protonix or angastra or apton or eupantol or inipomp or gastromax or noprop or pamgest or pantecta or panto or pantoc or pantocal or pantocarm or pantodac or pantop or pantopan or pantopaz or pantorc or pantozol or (pantozol adj rifun) or pantus or peptazol or protium or rifun or singastril or somac or supracam or ulcemex or ulcotenal or ulserch or ziprol or zurcal or zurcale or zurcazol or aciphex or gastrodine or pariet or rabec or rabeloc).ti,ab,rn. | 32970 |
| 2 | (inappropriate or overtreat* or overuse or overutil* or unnecessary or obsole* or 'low value' or low-value or wasteful or de-adopt* or deadopt* or deprescript* or deprescrib* or de-implement* or deimplement* or discontinu* or reduc* or stop* or withdraw* or abandon* or avoid or minimi* or decreas* or disinvest* or replac* or decommission* or decline).ti,ab,kf. | 5916445 |
| 3 | exp Deprescriptions/ | 309 |
| 4 | exp Inappropriate Prescribing/ | 2981 |
| 5 | exp Medical Overuse/ | 6930 |
| 6 | 3 or 4 or 5 | 10076 |
| 7 | 2 or 6 | 5920738 |
| 8 | 1 and 7 | 10890 |
| 9 | exp Hospitalization/ | 230989 |
| 10 | Inpatients/ | 20860 |
| 11 | exp Secondary Care/ | 573 |
| 12 | exp Tertiary Healthcare/ | 996 |
| 13 | ((second* or tertiary) adj2 care).ti,ab,kf. | 55991 |
| 14 | (hospitali* or inpatient* or ((second* or tertiary) adj2 care) or (hospital adj2 care)).ti,ab,kf. | 384534 |
| 15 | Adolescent, Hospitalized/ or hospitals/ | 76952 |
| 16 | (hospitali?ed or hospitali?ation or ward or wards).ti,ab. | 263805 |
| 17 | 9 or 10 or 11 or 12 or 13 or 14 or 15 or 16 | 630288 |
| 18 | 8 and 17 | 686 |

**Supplementary Table 2b. Embase search strategy sorted by search number ascending**

| No. | Query | Results |
| --- | --- | --- |
| #13 | #11 AND 'Article'/it AND [embase]/lim | 1578 |
| #12 | #11 AND 'Article'/it | 1628 |
| #11 | #7 AND #10 | 2904 |
| #10 | #8 OR #9 | 923865 |
| #9 | hospitali*:ti,ab OR inpatient*:ti,ab OR (((second* OR tertiary) NEAR/2 care):ti,ab) OR ((hospital NEAR/2 care):ti,ab) OR hospitali?ed:ti,ab OR hospitali?ation:ti,ab OR ward:ti,ab OR wards:ti,ab | 685488 |
| #8 | 'hospitalization'/exp OR 'hospital patient'/exp OR 'secondary health care'/exp OR 'tertiary health care'/exp | 582631 |
| #7 | #3 AND #6 | 29993 |
| #6 | #4 OR #5 | 8230716 |
| #5 | 'deprescription'/exp OR 'inappropriate prescribing'/exp | 5396 |
| #4 | inappropriate:ti,ab,de OR overtreat*:ti,ab,de OR overuse:ti,ab,de OR overutil*:ti,ab,de OR unnecessary:ti,ab,de OR obsole*:ti,ab,de OR 'low value':ti,ab,de OR wasteful:ti,ab,de OR 'de adopt*':ti,ab,de OR deadopt*:ti,ab,de OR deprescript*:ti,ab,de OR deprescrib*:ti,ab,de OR 'de implement*':ti,ab,de OR deimplement*:ti,ab,de OR discontinu*:ti,ab,de OR reduc*:ti,ab,de OR stop*:ti,ab,de OR withdraw*:ti,ab,de OR abandon*:ti,ab,de OR avoid:ti,ab,de OR minimi*:ti,ab,de OR decreas*:ti,ab,de OR disinvest*:ti,ab,de OR replac*:ti,ab,de OR decommission*:ti,ab,de OR decline:ti,ab,de | 8230716 |
| #3 | #1 OR #2 | 77596 |
| #2 | (('proton pump' NEXT/2 inhibitor?):ti,ab) OR ((rabeprazol?:ti,ab,rn,tn OR pantoprazol?:ti,ab,rn,tn OR lansoprazol?:ti,ab,rn,tn OR esomeprazol?:ti,ab,rn,tn OR antgra:ti,ab,rn,tn OR audazol:ti,ab,rn,tn OR aulcer:ti,ab,rn,tn OR belmazol:ti,ab,rn,tn OR ceprandal:ti,ab,rn,tn OR danlox:ti,ab,rn,tn OR demepraxol:ti,ab,rn,tn OR desec:ti,ab,rn,tn OR dizprazol:ti,ab,rn,tn OR dudencer:ti,ab,rn,tn OR elgam:ti,ab,rn,tn OR emeproton:ti,ab,rn,tn OR epirazole:ti,ab,rn,tn OR erbolin:ti,ab,rn,tn OR exter:ti,ab,rn,tn OR gasec:ti,ab,rn,tn OR gastrimut:ti,ab,rn,tn OR gastroloc:ti,ab,rn,tn OR gibancer:ti,ab,rn,tn OR indurgan:ti,ab,rn,tn OR inhibitron:ti,ab,rn,tn OR inhipump:ti,ab,rn,tn OR lensor:ti,ab,rn,tn OR logastric:ti,ab,rn,tn OR lomac:ti,ab,rn,tn OR losec:ti,ab,rn,tn OR mepral:ti,ab,rn,tn OR miol:ti,ab,rn,tn OR miracid:ti,ab,rn,tn OR mopral:ti,ab,rn,tn OR morecon:ti,ab,rn,tn OR nilsec:ti,ab,rn,tn OR nopramin:ti,ab,rn,tn OR amep:ti,ab,rn,tn OR omp:ti,ab,rn,tn OR omz:ti,ab,rn,tn OR ocid:ti,ab,rn,tn OR olexin:ti,ab,rn,tn OR omapren:ti,ab,rn,tn OR omed:ti,ab,rn,tn OR omegast:ti,ab,rn,tn OR omepral:ti,ab,rn,tn OR omeprazol:ti,ab,rn,tn OR omeprazole:ti,ab,rn,tn OR omeprazolum:ti,ab,rn,tn OR omeprazon:ti,ab,rn,tn OR omeprol:ti,ab,rn,tn OR omesek:ti,ab,rn,tn OR omezol:ti,ab,rn,tn OR omezolan:ti,ab,rn,tn OR omid:ti,ab,rn,tn OR omisec:ti,ab,rn,tn OR dakar:ti,ab,rn,tn OR omizac:ti,ab,rn,tn OR ompanyt:ti,ab,rn,tn OR ortanol:ti,ab,rn,tn OR osiren:ti,ab,rn,tn OR ozoken:ti,ab,rn,tn OR paprazol:ti,ab,rn,tn OR parizac:ti,ab,rn,tn OR pepticum:ti,ab,rn,tn OR pepticus:ti,ab,rn,tn OR peptilcer:ti,ab,rn,tn OR prazentol:ti,ab,rn,tn OR prazidec:ti,ab,rn,tn OR prazolit:ti,ab,rn,tn OR prilosec:ti,ab,rn,tn OR procelac:ti,ab,rn,tn OR proclor:ti,ab,rn,tn OR prysma:ti,ab,rn,tn OR ramezol:ti,ab,rn,tn OR regulacid:ti,ab,rn,tn OR sanamidol:ti,ab,rn,tn OR secrepina:ti,ab,rn,tn OR tedec:ti,ab,rn,tn) AND ulceral:ti,ab,rn,tn) OR ulceral:ti,ab,rn,tn OR ulcesep:ti,ab,rn,tn OR ulcometion:ti,ab,rn,tn OR ulcozol:ti,ab,rn,tn OR ulcsep:ti,ab,rn,tn OR ulsen:ti,ab,rn,tn OR ultop:ti,ab,rn,tn OR ulzol:ti,ab,rn,tn OR victrix:ti,ab,rn,tn OR zefxon:ti,ab,rn,tn OR zegerid:ti,ab,rn,tn OR zepral:ti,ab,rn,tn OR zimor:ti,ab,rn,tn OR zoltum:ti,ab,rn,tn OR zanprol:ti,ab,rn,tn OR ufiprazole:ti,ab,rn,tn OR ufiprazol:ti,ab,rn,tn OR ufiprazolum:ti,ab,rn,tn OR nexium:ti,ab,rn,tn OR perprazole:ti,ab,rn,tn OR nexiam:ti,ab,rn,tn OR inexium:ti,ab,rn,tn OR sompraz:ti,ab,rn,tn OR axagon:ti,ab,rn,tn OR esopral:ti,ab,rn,tn OR lucen:ti,ab,rn,tn OR axiago:ti,ab,rn,tn OR agopton:ti,ab,rn,tn OR alexin:ti,ab,rn,tn OR amarin:ti,ab,rn,tn OR aprazol:ti,ab,rn,tn OR bamalite:ti,ab,rn,tn OR blason:ti,ab,rn,tn OR compraz:ti,ab,rn,tn OR estomil:ti,ab,rn,tn OR fudermex:ti,ab,rn,tn OR gastrex:ti,ab,rn,tn OR gastride:ti,ab,rn,tn OR gastroliber:ti,ab,rn,tn OR ilsatec:ti,ab,rn,tn OR ketian:ti,ab,rn,tn OR keval:ti,ab,rn,tn OR lancid:ti,ab,rn,tn OR lanfast:ti,ab,rn,tn OR lanproton:ti,ab,rn,tn OR lansopep:ti,ab,rn,tn OR lansoprazolum:ti,ab,rn,tn OR lansox:ti,ab,rn,tn OR lanston:ti,ab,rn,tn OR lanz:ti,ab,rn,tn OR lanzo:ti,ab,rn,tn OR lanzogastro:ti,ab,rn,tn OR lanzol:ti,ab,rn,tn OR lanzopral:ti,ab,rn,tn OR lanzor:ti,ab,rn,tn OR lasoprol:ti,ab,rn,tn OR limpidex:ti,ab,rn,tn OR lizul:ti,ab,rn,tn OR mesactol:ti,ab,rn,tn OR monolitum:ti,ab,rn,tn OR ogast:ti,ab,rn,tn OR ogasto:ti,ab,rn,tn OR ogastro:ti,ab,rn,tn OR opiren:ti,ab,rn,tn OR pampe:ti,ab,rn,tn OR peptomil:ti,ab,rn,tn OR prevacid:ti,ab,rn,tn OR prezal:ti,ab,rn,tn OR promp:ti,ab,rn,tn OR prosogan:ti,ab,rn,tn OR suprecid:ti,ab,rn,tn OR takepron:ti,ab,rn,tn OR ulcertec:ti,ab,rn,tn OR uldapril:ti,ab,rn,tn OR ulpax:ti,ab,rn,tn OR unival:ti,ab,rn,tn OR zoprol:ti,ab,rn,tn OR zoton:ti,ab,rn,tn OR pantoprazol:ti,ab,rn,tn OR pantoprazole:ti,ab,rn,tn OR pantoprazolum:ti,ab,rn,tn OR controloc:ti,ab,rn,tn OR pantoloc:ti,ab,rn,tn OR protonix:ti,ab,rn,tn OR angastra:ti,ab,rn,tn OR apton:ti,ab,rn,tn OR eupantol:ti,ab,rn,tn OR inipomp:ti,ab,rn,tn OR gastromax:ti,ab,rn,tn OR noprop:ti,ab,rn,tn OR pamgest:ti,ab,rn,tn OR pantecta:ti,ab,rn,tn OR panto:ti,ab,rn,tn OR pantoc:ti,ab,rn,tn OR pantocal:ti,ab,rn,tn OR pantocarm:ti,ab,rn,tn OR pantodac:ti,ab,rn,tn OR pantop:ti,ab,rn,tn OR pantopan:ti,ab,rn,tn OR pantopaz:ti,ab,rn,tn OR pantorc:ti,ab,rn,tn OR pantozol:ti,ab,rn,tn OR (pantozol:ti,ab,rn,tn AND adj:ti,ab,rn,tn AND rifun:ti,ab,rn,tn) OR pantus:ti,ab,rn,tn OR peptazol:ti,ab,rn,tn OR protium:ti,ab,rn,tn OR rifun:ti,ab,rn,tn OR singastril:ti,ab,rn,tn OR somac:ti,ab,rn,tn OR supracam:ti,ab,rn,tn OR ulcemex:ti,ab,rn,tn OR ulcotenal:ti,ab,rn,tn OR ulserch:ti,ab,rn,tn OR ziprol:ti,ab,rn,tn OR zurcal:ti,ab,rn,tn OR zurcale:ti,ab,rn,tn OR zurcazol:ti,ab,rn,tn OR aciphex:ti,ab,rn,tn OR gastrodine:ti,ab,rn,tn OR pariet:ti,ab,rn,tn OR rabec:ti,ab,rn,tn OR rabeloc:ti,ab,rn,tn | 18795 |
| #1 | 'proton pump inhibitor'/exp | 75825 |

**Supplementary Table 3. Definitions of interventions of the included studies based on EPOC taxonomy for implementation strategies**^(^[^8^](#_ENREF_8)^)^

| **Category** | **definition** |
| --- | --- |
| Audit and feedback | A summary of health workers’ performance over a specified period of time, given to them in a written, electronic or verbal format. The summary may include recommendations for clinical action. |
| Clinical Practice Guidelines | Clinical guidelines are systematically developed statements to assist healthcare providers and patients to decide on appropriate health care for specific clinical circumstances'(US IOM). |
| Educational materials | Distribution to individuals, or groups, of educational materials to support clinical care, i.e., any intervention in which knowledge is distributed. For example, this may be facilitated by the internet, learning critical appraisal skills; skills for electronic retrieval of information, diagnostic formulation; question formulation |
| Educational meetings | Courses, workshops, conferences or other educational meetings |
| Inter-professional education | Continuing education for health professionals that involves more than one profession in joint, interactive learning |
| Local opinion leaders | The identification and use of identifiable local opinion leaders to promote good clinical practice. |
| Organizational culture | Strategies to change organizational culture |
| Reminders | Manual or computerized interventions that prompt health workers to perform an action during a consultation with a patient, for example computer decision support systems. |

**Supplementary Table 4. Appropriate use of PPI for SUP according to included studies**

| **Medical condition** | **Symptoms or risk factors** | **References** |
| --- | --- | --- |
| Peptic ulcer disease (PUD) | - Following endoscopy therapy for PUD or endoscopy diagnostic procedures - Gastric or duodenal ulcer treatment/ healing - Treatment or history (within 1 year) of gastrointestinal (GI) disorders or GI bleeding | ([1](#_ENREF_1), [3](#_ENREF_3), [4](#_ENREF_4), [7](#_ENREF_7), [9-11](#_ENREF_9)) |
| Gastroesophageal reflux disease (GERD) | - Treatment or history (within 1 year) of GI disorders or GI bleeding - Prophylaxis of acid aspiration | ([1](#_ENREF_1), [3](#_ENREF_3), [4](#_ENREF_4), [9-11](#_ENREF_9)) |
| Prevention of PUD/ non-steroidal anti-inflammatory drugs (NSAID)-associated ulcers | Presence of (at least) of the following two risk factors:   - Prevention of side effects of a combination of medications:   - NSAID (long term or high dose) or low dose aspirin  - antiplatelet agents/ anti-aggregants  - (high dose) corticosteroids  - anticoagulant drugs (e.g. warfarin or heparin)  - selective serotonin re-uptake inhibitors (SSRI)   - Age > 60 years - History of GI bleeding or PUD or *Helicobacter pylori* infection - Respiratory failure without intubation - Heart failure requiring inotropic support - Sepsis - Stroke - Hepatic failure (e.g. hepatic encephalopathy or jaundice) - Renal failure - Hypotension - Occult bleeding (>6 days) | ([1](#_ENREF_1), [2](#_ENREF_2), [5](#_ENREF_5), [9-11](#_ENREF_9)) |
| Zollinger-Ellison Syndrome |  | ([1](#_ENREF_1), [9](#_ENREF_9)) |
| Eradication of *H. pylori* |  | ([1](#_ENREF_1), [9](#_ENREF_9), [11](#_ENREF_11)) |
| (Risk) of upper GI bleeding | - Coagulopathy (platelet count<50000) or International Normalized Ratio (INR)>1.5 or Partial Thromboplastin Time (PTT) >2 time normal - Liver cirrhosis | ([1](#_ENREF_1), [4](#_ENREF_4), [9](#_ENREF_9)) |
| Other | - Organ transplantation | ([9](#_ENREF_9)) |

**Supplementary Table 5. Definition of SUP in the included studies**

| **Source** | **Evidence or definition of inappropriate PPI use** | | **Definition specifically described** |
| --- | --- | --- | --- |
| del Giorno 2018 | literature, guideline | regional/ national guidelines, Uptodate, ([12](#_ENREF_12), [13](#_ENREF_13)) | yes |
| Jain 2013 | literature | ([14](#_ENREF_14)) | yes |
| Kehr 2011 | guideline | ([15](#_ENREF_15)) | yes |
| Khalili 2010 | guideline | ([15](#_ENREF_15)) | yes |
| Khudair 2011 | literature | ([15-20](#_ENREF_15)) | yes |
| Kumana 1998 | single disciplinary panel | relevant specialists | yes |
| Luo 2018 | guideline | national guideline 2015 ([21](#_ENREF_21)) | no |
| van Vliet 2009 | literature, guideline | national guidelines 2003, 2004, ([22-28](#_ENREF_22)) | yes |
| Xin 2018 | literature, guideline | national guideline 2014, ([29](#_ENREF_29)) | no |
| Yachimski 2010 | literature, multidisciplinary panel | consensus set of guidelines based on literature | yes |

*) implementation of an institutional clinical guideline

**Supplementary Table 6. Detailed information on risk of bias assessment**

**Risk of bias table – Del Giorno 2018**

| **Item** | **Judgment*** | **Description** |
| --- | --- | --- |
| Random sequence generation? | high | Participants were not randomized. |
| Allocation concealment? | high | Allocation was not concealed |
| Baseline outcomes similar? | unclear | No information provided |
| Free of contamination? | unclear | It is unclear whether providers from the internal medicine department have influenced health care providers of the surgery department. |
| Blinding of outcome assessment? | unclear | No information provided |
| Incomplete outcome data addressed? | low | All inpatients analyzed; no missing outcome data reported (chart review) |
| Baseline characteristics similar? | high | The intervention group is the internal medicine department and the control group is the surgery departments. Although age and gender are similar, characteristics of both patients and providers is different. |
| Intervention independent of other changes? | unclear | Stepwise implementation of period of 4 years. No further information provided |
| Intervention unlikely to affect data collection? | low | Sources and methods of data collection are assumed the same before and after the intervention (chart review) |
| Free of selective reporting? | unclear | No study protocol available |
| Free of other bias? | low | No evidence of other risks of bias |

**Risk of bias table – Jain 2013**

| **Item** | **Judgment*** | **Description** |
| --- | --- | --- |
| Random sequence generation? | NA |  |
| Allocation concealment? | NA |  |
| Baseline outcomes similar? | NA |  |
| Free of contamination? | NA |  |
| Blinding of outcome assessment? | unclear | No information provided |
| Incomplete outcome data addressed? | unclear | No information provided |
| Baseline characteristics similar? | unclear | No information provided |
| Intervention independent of other changes? | unclear | No information provided |
| Intervention unlikely to affect data collection? | low | Same standardized data extraction form before and after intervention used. |
| Free of selective reporting? | unclear | No study protocol available |
| Free of other bias? | low | No evidence of other risks of bias |

**Risk of bias table – Kehr 2011**

| **Item** | **Judgment*** | **Description** |
| --- | --- | --- |
| Random sequence generation? | NA |  |
| Allocation concealment? | NA |  |
| Baseline outcomes similar? | NA |  |
| Free of contamination? | NA |  |
| Blinding of outcome assessment? | unclear | No information provided |
| Incomplete outcome data addressed? | low | No missing outcome data reported (chart review) |
| Baseline characteristics similar? | low | Demographic analysis showed no significant difference between baseline and post-implementation with relation to patient age or gender. At all timepoints the majority of the patients is in the general ward (95%, 90%, 92%). |
| Intervention independent of other changes? | low | Relative short time period for intervention, unlikely to be influenced. |
| Intervention unlikely to affect data collection? | low | Standardized data-extraction during all three periods. |
| Free of selective reporting? | unclear | No study protocol available |
| Free of other bias? | low | No evidence of other risks of bias |

**Risk of bias table – Khalili 2010**

| **Item** | **Judgment*** | **Description** |
| --- | --- | --- |
| Random sequence generation? | NA |  |
| Allocation concealment? | NA |  |
| Baseline outcomes similar? | NA |  |
| Free of contamination? | NA |  |
| Blinding of outcome assessment? | unclear | No information provided |
| Incomplete outcome data addressed? | low | Small amount of patients is lost to follow-up, and this is balanced between pre-intervention and post-intervention group (fig 2) |
| Baseline characteristics similar? | high | Age and gender are similarly distributed between pre-intervention and post-intervention group, but risk factors in patients are not similar in pre-intervention and post-intervention period (heparin 19.5% vs 14.6%; NSAID use for more than 3 months 10.7% vs 7.1%; corticosteroid use 6.1% vs 2.7%) |
| Intervention independent of other changes? | low | Relative short time period for intervention, unlikely to be influenced. |
| Intervention unlikely to affect data collection? | unclear | Not specified how data is collected |
| Free of selective reporting? | unclear | No study protocol available |
| Free of other bias? | low | No evidence of other risks of bias |

**Risk of bias table – Khudair 2011**

| **Item** | **Judgment*** | **Description** |
| --- | --- | --- |
| Random sequence generation? | NA |  |
| Allocation concealment? | NA |  |
| Baseline outcomes similar? | NA |  |
| Free of contamination? | NA |  |
| Blinding of outcome assessment? | unclear | No information available |
| Incomplete outcome data addressed? | unclear | No information available |
| Baseline characteristics similar? | low | Age, gender and ethnicity are similarly distributed between pre-intervention and post-intervention group |
| Intervention independent of other changes? | low | 1 year between intervention and follow-up;  “ 1 year gap was likely to reflect the real impact of the multi-approach strategy, allowing the initial enthusiasm of following the institutional guidelines to settle. Audit 2 was conducted in the exact same months as Audit 1. This would limit a possible variation in the seasonal influx of patients” |
| Intervention unlikely to affect data collection? | low | Similar data-collection form was used during both audits |
| Free of selective reporting? | unclear | No study protocol available |
| Free of other bias? | low | No evidence of other risks of bias |

**Risk of bias table – Kumana 1998**

| **Item** | **Judgment*** | **Description** |
| --- | --- | --- |
| Random sequence generation? | NA |  |
| Allocation concealment? | NA |  |
| Baseline outcomes similar? | NA |  |
| Free of contamination? | NA |  |
| Blinding of outcome assessment? | unclear | No information provided |
| Incomplete outcome data addressed? | low | Successful follow-up was not achieved for 91 of the patients for whom memoranda were sent because they were either discharged or dead. Among those who were followed up, inappropriate prescribing continued without justification and despite reminders in only 6 instances. |
| Baseline characteristics similar? | unclear | No information provided |
| Intervention independent of other changes? | low | “correspond drug usage data for other hospitals in the territory, which were not implementing specific strategies directed at antiulcer drug usage at that time, was also retrieved from the computerized pharmaceutical supplies system of the Hong Kong Hospital Authority” No effect on PPI prescriptions was observed in these hospitals in the period corresponding to the post-intervention period in the hospital involved in the study. |
| Intervention unlikely to affect data collection? | unclear | Data were obtained from the hospital pharmacy, no specific information available how data were collected |
| Free of selective reporting? | unclear | No study protocol available |
| Free of other bias? | low | No evidence of other risks of bias |

**Risk of bias table – Luo 2018**

| **Item** | **Judgment*** | **Description** |
| --- | --- | --- |
| Random sequence generation? | NA |  |
| Allocation concealment? | NA |  |
| Baseline outcomes similar? | NA |  |
| Free of contamination? | NA |  |
| Blinding of outcome assessment? | low | Automated data collection, stated to be anonymised |
| Incomplete outcome data addressed? | low | No missing outcome data reported (retrospective chart review) |
| Baseline characteristics similar? | low | Age, gender and BMI are similarly distributed between pre-intervention and post-intervention group. Patients are randomly (computer-based randomisation) selected from all patient charts. |
| Intervention independent of other changes? | low | Relative short time period for intervention, unlikely to be influenced. (Jul-Dec 2015 pre-intervention; Jul-Dec2016 post-intervention) |
| Intervention unlikely to affect data collection? | low | “The Hospital Information System and Prescription Automatic Screening System of Sichuan Medico Software Research and Development Co., Ltd., were used to collect the numbers of patients in outpatient and inpatient settings and the data related to PPI application including drug name, dosage form, specification, unit price, volume, and manufacturer from 2007 to 2016. All data collected was anonymized and could not be traced back to an individual. The rates of PPI prescribing, defined daily doses.  (DDDs), DDDs/1000 inhabitants per day (DDDs/TID) and expenditure were used for measuring drug utilization and expenditure, which was in line with international recommendations. |
| Free of selective reporting? | unclear | No study protocol available |
| Free of other bias? | low | No evidence of other risks of bias |

**Risk of bias table – van Vliet 2009**

| **Item** | **Judgment*** | **Description** |
| --- | --- | --- |
| Random sequence generation? | NA |  |
| Allocation concealment? | NA |  |
| Baseline outcomes similar? | NA |  |
| Free of contamination? | NA |  |
| Blinding of outcome assessment? | Unclear | Unclear how post-intervention data were collected. |
| Incomplete outcome data addressed? | low | No missing data |
| Baseline characteristics similar? | low | Age, gender are similarly distributed between pre-intervention and post-intervention group. The percentage of cardiovascular disease patients is different, but all other clinical parameters are similar between the two groups. |
| Intervention independent of other changes? | low | Short time period for intervention, unlikely to be influenced.( before Oct 2004-April 2005 and after June 2005-February 2006). |
| Intervention unlikely to affect data collection? | low | Data collection was done the same way as was done in the first study period |
| Free of selective reporting? | unclear | No study protocol available |
| Free of other bias? | low | No evidence of other risks of bias |

**Risk of bias table – Xin 2018**

| **Item** | **Judgment*** | **Description** |
| --- | --- | --- |
| Random sequence generation? | NA |  |
| Allocation concealment? | NA |  |
| Baseline outcomes similar? | NA |  |
| Free of contamination? | NA |  |
| Blinding of outcome assessment? | unclear | No information available |
| Incomplete outcome data addressed? | low | No missing data |
| Baseline characteristics similar? | low | Age, gender are similarly distributed between pre-intervention and post-intervention group. Most clinical parameters are similar between the two groups. |
| Intervention independent of other changes? | low | Short time period for intervention end follow-up, unlikely to be influenced.(3-9 months) |
| Intervention unlikely to affect data collection? | low | Data collection was done the same way as was done in the first study period  “The medical information of patients was identified through the Computerized Patient Record System, which listed all the information of inpatients who had received PPI therapy.”  Data on drug therapy were collected as follows: diagnoses, categories of PPIs therapy indication, dosage, administration rout, costs, duration of therapy and comorbidities. |
| Free of selective reporting? | unclear | No study protocol available |
| Free of other bias? | low | No evidence of other risks of bias |

**Risk of bias table – Yachimski 2010**

| **Item** | **Judgment*** | **Description** |
| --- | --- | --- |
| Random sequence generation? | NA |  |
| Allocation concealment? | NA |  |
| Baseline outcomes similar? | NA |  |
| Free of contamination? | NA |  |
| Blinding of outcome assessment? | low | Outcome data were retrievable through search of computerized provider order entry. |
| Incomplete outcome data addressed? | low | No missing data |
| Baseline characteristics similar? | low | Age, gender are similarly distributed between pre-intervention and post-intervention group. Most clinical parameters are similar between the two groups. |
| Intervention independent of other changes? | low | Short time period for intervention end follow-up, unlikely to be influenced.(1 month) |
| Intervention unlikely to affect data collection? | low | “Massachusetts General Hospital uses an electronic medical record, and provider order entry is computer based. We extracted demographic data, including age and sex, from the electronic medical record. Medical history, including history of  gastroesophageal reflux disease, peptic ulcer disease, or UGIB, and outpatient medication use were defined as documented by the house staff in the history and physical at admission. The study defined inpatient PPI use as the presence of a physician’s order for formulary PPIs at any point during a patient’s hospital admission, retrievable through a search of computerized provider order entry. Proton pump inhibitor use at discharge was defined as the inclusion of a prescription for PPIs among the patient’s discharge medications in the electronic discharge summary |
| Free of selective reporting? | unclear | No study protocol available |
| Free of other bias? | low | No evidence of other risks of bias |

**References**

1. Del Giorno R, Ceschi A, Pironi M, Zasa A, Greco A, Gabutti L. Multifaceted intervention to curb in-hospital over-prescription of proton pump inhibitors: A longitudinal multicenter quasi-experimental before-and-after study. European Journal of Internal Medicine. 2018;50:52-9. doi:10.1016/j.ejim.2017.11.002

2. Jain G, Jabeen S, Vallurupalli S. Efforts to Reduce Stress Ulcer Prophylaxis Use

in Non-Critically Ill Hospitalized Patients by Internal Medicine Residents: A Single-Institution Experience. JCOM. 2013;20(1):13-9.

3. Kehr H, CL G, Haynes R, S E, BH M. Evaluation of Stress Ulcer Prophylaxis in a Family Medicine Residency Inpatient Service. JCOM. 2011;18(3):102-6.

4. Khalili H, Dashti-Khavidaki S, Hossein Talasaz AH, Tabeefar H, Hendoiee N. Descriptive analysis of a clinical pharmacy intervention to improve the appropriate use of stress ulcer prophylaxis in a hospital infectious disease ward. J Manage Care Pharm. 2010;16(2):114-21. doi:10.18553/jmcp.2010.16.2.114

5. Luo H, Fan Q, Xiao S, Chen K. Changes in proton pump inhibitor prescribing trend over the past decade and pharmacists' effect on prescribing practice at a tertiary hospital. BMC Health Serv Res. 2018;18(1):537. doi:10.1186/s12913-018-3358-5

6. Xin C, Dong Z, Lin M, Li GH. The impact of pharmaceutical interventions on the rational use of proton pump inhibitors in a Chinese hospital. Patient Prefer Adherence. 2018;12:21-6. doi:10.2147/PPA.S150388

7. Yachimski PS, Farrell EA, Hunt DP, Reid AE. Proton pump inhibitors for prophylaxis of nosocomial upper gastrointestinal tract bleeding: effect of standardized guidelines on prescribing practice. Arch Intern Med. 2010;170(9):779-83. doi:10.1001/archinternmed.2010.51

8. EffectivePracticeandOrganisationofCare(EPOC). EPOC Taxonomy. epoc.cochrane.org/epoc-taxonomy. 2015. 2020.

9. Khudair IF, Sadik ND, Hanssens Y, Muhsin SA, Matar I. Impact of multi-approach strategy on acid suppressive medication use in a teaching hospital in Qatar. Int J Clin Pharm. 2011;33(5):763-71. doi:10.1007/s11096-011-9531-0

10. van Vliet EP, Steyerberg EW, Otten HJ, Rudolphus A, Knoester PD, Hoogsteden HC, et al. The effects of guideline implementation for proton pump inhibitor prescription on two pulmonary medicine wards. Aliment Pharmacol Ther. 2009;29(2):213-21. doi:10.1111/j.1365-2036.2008.03875.x

11. Kumana CR, Ching TY, Cheung E, Kong Y, Kou M, Chan CK, et al. Antiulcer drug prescribing in hospital successfully influenced by "immediate concurrent feedback". Clin Pharmacol Ther. 1998;64(5):569-74. doi:10.1016/S0009-9236(98)90141-2

12. Schoenfeld A, Grady D. Adverse effects assosiated tih Proton Pump Inhibitors. JAMA Intern Med. 2016;176(2):172-4.

13. Gomm W, von Holt K, Thome F, Broich K, Maier W, Fink A, et al. Association of Proton Pump Inhibitors With Risk of DementiaA Pharmacoepidemiological Claims Data Analysis. Jama Neurology. 2016;73(4):410-6.

14. Estruch R, Pedrol E, Castells A, Masanes F, Marrades RM, Urbano-Marquez A. Prophylaxis of gastrointestinal tract bleeding with magaldrate in patients admitted to a general hospital ward. Scand J Gastroenterol. 1991;26(8):819-26. doi:10.3109/00365529109037018

15. ASHP. ASHP Therapeutic Guidelines on Stress Ulcer Prophylaxis. ASHP Commission on Therapeutics and approved by the ASHP Board of Directors on November 14, 1998. Am J Health Syst Pharm. 1999;56(4):347-79. doi:10.1093/ajhp/56.4.347

16. Leontiadis G, VK S, Howden C. Systematic Review and meta-analysis of proton pump inhibitor therapy in peptic ulcer bleeding. . BMJ. 2005;330:568-.

17. Skala I, Mareckova O, Vitko S, Matl I, Lacha J. Porphylaxis of acute gastroduodenal bleeding after renal transplantation. Transpl Int. 1997;10:371-8.

18. Chen LS, Lin HC, Hwang SJ, Lee FY, Hou MC, Lee SD. Prevalence of gastric ulcer in cirrhotic patients and its relation to portal hypertension. J Gastroenterol Hepatol. 1996;11(1):59-64. doi:10.1111/j.1440-1746.1996.tb00011.x

19. Piper J, Ray W, Daugherty J, Griffin M. Corticosteriod use and peptic ulcer disease: role of non-steroidal anti-inflammatory drugs. . Ann Intern Med. 1991;114(735-740).

20. Lanza F. A guideling for the treatment and prevention of NSAID-inuced ulcers. . Am J Gastroenterol. 1998;93:2037-46.

21. Ye ZK, Liu Y, Cui XL, Liu LH. Critical Appraisal of the Quality of Clinical Practice Guidelines for Stress Ulcer Prophylaxis. PLoS One. 2016;11(5):e0155020. doi:10.1371/journal.pone.0155020

22. Richardson P, Hawkey CJ, Stack WA. Proton pump inhibitors. Pharmacology and rationale for use in gastrointestinal disorders. Drugs. 1998;56(3):307-35. doi:10.2165/00003495-199856030-00002

23. Triadifilopoulus G. Proton pump inhibitors for Barett’s Esophagus. Gut. 2000;46:144-6.

24. Vanderhoff B, Tahhoub R. Proton Pump Inhibitors; an update. . Am Fam Physician. 2002;66:273-80.

25. Numans M, De Wit N, Dirven J, Hurenkamp G, Meijet Q, Muris J. NHG standard Maagklachten. Huisarts Wet. 2003;46:690-700.

26. Chan F, DY G. Review article: prevention of non-steroidal anti-inflammatory drug gastroentestinal complications-review and receommendations based on risk assessment. Aliment Pharmacol Ther. 2004;19:1051-61.

27. Dubois R, Melmed G, Henning J, Laine L. Guidelines for the appropriate use of non-steroidal anti-inflammatory drugs, cyclo-oxygenase-2-specific inhibitors and proton pump inhibitors in patients requiring chronic anti-inflammatory therapy. . Aliment Pharmacol Ther. 2004;19:197-208.

28. Zullo A, Hassan C, Campo S, Morini S. Bleeding peptic ulcer in the elderly: risk factors and prevention strategies. . Drugs and aging. 2007;24:815-28.

29. Farrell B, Pottie K, Thompson W, Boghossian T, Pizzola L, Rashid FJ, et al. Deprescribing proton pump inhibitors: Evidence-based clinical practice guideline. Can Fam Physician. 2017;63(5):354-64.
